# Comparison of rRNA depletion methods for efficient bacterial mRNA sequencing

**DOI:** 10.1038/s41598-022-09710-y

**Published:** 2022-04-06

**Authors:** Anika Wahl, Christopher Huptas, Klaus Neuhaus

**Affiliations:** 1 Core Facility Microbiome, ZIEL – Institute for Food and Health, Technische Universität München, Weihenstephaner Berg 3, 85354 Freising, Germany; 2 Chair for Microbial Ecology, Technische Universität München, Weihenstephaner Berg 3, 85354 Freising, Germany

**Keywords:** Bacterial transcription, Transcription, Translation

## Abstract

Current methods of high-throughput RNA sequencing of prokaryotes, including transcriptome analysis or ribosomal profiling, need deep sequencing to achieve sufficient numbers of effective reads (e.g., mapping to mRNA) in order to also find weakly expressed genetic elements. The fraction of high-quality reads mapping to coding RNAs (i.e., mRNA) is mainly influenced by the large content of rRNA and, to a lesser extent, tRNA in total RNA. Thus, depletion of rRNA increases coverage and thus sequencing costs. RiboZero, a depletion kit based on probe hybridisation and rRNA-removal was found to be most efficient in the past, but it was discontinued in 2018. To facilitate comparability with previous experiments and to help choose adequate replacements, we compare three commercially available rRNA depletion kits also based on hybridization and magnetic beads, i.e., riboPOOLs, RiboMinus and MICROBExpress, with the former RiboZero. Additionally, we constructed biotinylated probes for magnetic bead capture and rRNA depletion in this study. Based on *E. coli*, we found similar efficiencies in rRNA depletion for riboPOOLs and the self-made depletion method; both comparable to the former RiboZero, followed by RiboMinus, succeeded by MICROBExpress. Further, our in-house protocol allows customized species-specific rRNA or even tRNA depletion or depletion of other RNA targets. Both, the self-made biotinylated probes and riboPOOLs, were most successful in reducing the rRNA content and thereby increasing sequencing depth concerning mRNA reads. Additionally, the number of reads matching to weakly expressed genes are increased. In conclusion, the self-made specific biotinylated probes and riboPOOLs are an adequate replacement for the former RiboZero. Both are very efficient in depleting rRNAs, increasing mRNA reads and thus sequencing efficiency.

## Introduction

High-throughput sequencing of mRNA or ribosomal profiling provides detailed insight into cellular transcriptomes and translatomes^1–3^. However, just sequencing total RNA is ineffective, since most reads will map to rRNA or tRNA. For example, bacterial total RNA contains at least 80–90% rRNA when harvested during exponential growth, which limits data output^4–6^. While RNA-seq without previous depletion of rRNA have been conducted using deep sequencing (i.e., brute force), investigation of weakly expressed mRNAs or regulatory RNAs is still limited^7–9^. Thus, depletion of rRNA is needed to enrich the fraction of desired mRNA reads and thereby increase their coverage. For instance, a sequencing depth of about 2 million effective reads (i.e., reads mapping mRNA) seems to be sufficient for analysing gene regulation of annotated genes in RNA sequencing (RNA-seq;^10^), whereas about 20 million effective reads are suggested for ribosome-profiling assisted detection of novel protein-coding ORFs and analysing weakly expressed genes^11^. While sequencing prices are still dropping slightly, a plateau has been reached^12–15^ and still, more useful data is generated for less money if mRNA is enriched by rRNA depletion.

Three major strategies to achieve rRNA depletion are used. The first is based on the hybridization of oligonucleotides to rRNA and subsequent capture and separation with magnetic beads^16–19^. The second is based on annealing of complementary oligonucleotides and enzymatic digestion of the duplex hybrids by specific nucleases^20,21^. The third method limits cDNA synthesis to non-rRNA templates using a non-random primer mixture in cDNA production devoid of those matching to rRNA^22^.

In any case, the first strategy is most commonly used and shows consistent results in depleting rRNA without introducing major bias. Until November 2018, the RiboZero Gold rRNA depletion Kit from Illumina was available, which was superior in rRNA depletion according to several publications^19,23–29^. Its method was based on the first technique mentioned. However, the production of this kit was discontinued. In 2020, the kit re-entered the market with the same name, but with a substantial change in method. Now it is based on enzymatic digestion of rRNA hybridization complexes (i.e., second strategy). Interestingly, for this method a bias was reported when compared to sequencing of undepleted total RNA^28,30^. This bias seems to be even stronger in ribosome profiling^28^. Some of the nucleases used here seem to have off-target activity. Thus, results about ribosome position and information about the A-site status can be blurred in final results. Furthermore, both 5’ and 3’ ends of mRNA fragments can be digested unspecifically^28,31,32^. The complete change in methods makes comparison of sequencing results obtained using RiboZero before and after 2019 more difficult. To provide scientists with an informed choice, we compared the previous RiboZero (RZ, throughout the publication) with three other commercially available rRNA depletion kits: RiboMinus Transcriptome Isolation Kit, bacteria (RM; Thermo Fisher); MICROBExpress Bacterial mRNA Enrichment Kit (ME; Invitrogen), and riboPOOLs (RP; siTOOLs). Furthermore, we constructed biotinylated probes (designated BP) for a species-specific depletion method following the patent US 2011/0,040,081 A1^33^. All five depletion protocols are based on hybridization and magnetic bead capture. For comparability, we only chose this depletion strategy. The oligonucleotides used in RM, RP, and ME are short DNA probes of 20–30 nt antisense to the respective rRNAs. Additionally, it is known that the oligonucleotides contained in RM and ME do not cover the full length of the 16S rRNA^34–38^. In contrast, oligonucleotides used in RZ are antisense rRNA sequences covering the entire length of the rRNAs. Further, RM and ME target 16S and 23S rRNA but not 5S rRNA, whereas RP and RZ capture 5S rRNA as well. In order to provide the possibility of effective physical capture and (magnetic) depletion, the rRNA-targeting probes are labelled. All kits, except ME, use biotin, either at the 5’-end, the 3’-end or internally by introducing biotin-labelled UTP. Biotin is bound by streptavidin, which is the strongest non-covalent biological binding known^39^. Thus, magnetic beads covered with streptavidin are used for depletion. In contrast, probes in ME are polyA-tailed and, captured by poly-dT covered magnetic beads^34,35,40^. A short overview of the properties and differences for each kit is given in Supplementary table S1. Additionally, Supplementary table S2 summarizes former publications comparing additional kits for informed choices^41–43^. In any case, concerning the kits tested here, the oligonucleotides in the kits RM, ME, and the former RZ, are designed to hybridize to many different bacterial rRNAs. They are pan-prokaryotic in their target rRNAs. For RP, in the beginning, only a species-specific version was available. Thus, the *Escherichia coli* specific edition of the RP kit is used in this study. Meanwhile, a broader range of different variations is available for the RP kit, pan-prokaryotic and several species-specific editions. However, our self-designed oligonucleotides are species specific for *E. coli* following the patent, which is the basis for the former RZ. Furthermore, the probes of the ME and RZ were developed in the beginning on the basis of *E. coli* rRNA^31,33^. Finally, as stated in the protocols for RM and ME, quality control of depletion success is performed using total RNA isolated from *E. coli*. We assume, therefore, that all commercial kit should be optimized for *E. coli* RNA. Consequently, this model organism was chosen to compare the depletion kits in this study.

In this study, we aimed to find a suitable replacement for the former RiboZero to preserve comparability to previous experiments. Further, new kits like RP, have entered the market and were compared to RZ and a self-made method using RZ´s principle to determine the best rRNA depletion method(s) available. Fully optimized depletion of rRNA (and perhaps tRNAs) allows reducing costs in sequencing experiments like RNA-seq and Ribo-seq, while retaining the ability to get high-quality mRNA expression data.

## Methods

### Bacteria and primer used

*E. coli* TOP10 (Invitrogen) and *E. coli* DSM 30083^ T^ (DSMZ, Braunschweig) were cultivated at 37 °C in LB medium, either overnight for DNA isolation or until OD_600nm_ = 0.6 and 1.0 for RNA isolation. Cells were harvested at 12,000 × g at 4 °C for 5 min.

All primer used are listed in Supplementary table S3.

### DNA and RNA isolation

In all protocols, RNA and DNA concentrations were either determined by the NanoDrop 1000 spectrometer (Thermo Fisher Scientific) and/or by the Qubit 2.0 Fluorometer (Invitrogen, life technologies) according to the Qubit RNA HS Assay Kits and Qubit dsDNA HS Assay Kit. Genomic DNA was isolated according to a CTAB (cetyltrimethylammonium bromide)-based protocol as described ^44^. For RNA isolation, cell pellets were resuspended in 1 mL Trizol (Thermo Fisher) and mixed with approx. 0.2 mL 0.1-mm zirconia beads (Carl Roth, Germany). Cells were disrupted using the Ribolyzer (MP FastPrep) for bead beating (thrice at 6.5 m/s for 45 s). After incubation for 5 min at room temperature (RT), 0.2 volumes chloroform per mL Trizol were added and mixed for 15 s, followed by centrifugation at 12,000 × g at 4 °C for 15 min. The upper aqueous phase was transferred into new tubes and RNA was precipitated with isopropanol (Carl Roth). The RNA pellet was carefully dried at RT and solved in RNase-free water. Afterwards, DNA digestion was performed (Turbo DNase, life technologies). Complete DNA removal was tested with PCR using *Taq* DNA polymerase (Supplementary table S4 with primer rrsHR and rrsHF. Total RNA quality was checked using agarose gel electrophoresis and using the Bioanalyzer with the Agilent RNA 6000 Nano Kit (Agilent).

### Biotinylated antisense rRNA oligonucleotides

The former RiboZero was based on patent US 2011/0,040,081 A1^33^. Following this patent, primers amplifying the full length of 5S, 16S and 23S rDNA were selected. Since, the 23S rRNA is around 2,700 bp, the first and second part of this gene (designated 5′ 23S and 3′ 23S) were amplified separately to ease amplification. For later in vitro transcription of complementary rRNA, the reverse primer included the sequence of the T7 promoter. All primers are listed in Supplementary table S3 and the PCR program is enlisted in Supplementary table S4.

In contrast to the patent, we started with gDNA of the organism of choice instead of using reverse-transcribed rRNA, i.e., cDNA for final production of complementary rRNA probes. rRNA genes were amplified from gDNA in a PCR using Q5 High-Fidelity Polymerase (NEB; Supplementary table S5). PCR products were cleaned using the PCR Clean-up Kit (Thermo Scientific). To generate biotinylated complementary rRNA probes, in-vitro transcription was conducted. Accordingly, the AmpliScribe T7 High Yield Transcription Kit (BIOSEARCH TECHNOLOGIES, Lucigen) was used. Half of the UTP amount needed was substituted by biotin-16-UTP (Biozym). For each complementary rRNA probe (i.e., 5S, 16S, 5′ 23S, and 3′ 23S), 1 µg PCR-amplified rRNA gene product was used as template. Complementary rRNA probes were cleaned from template DNA by DNase I (NEB) treatment. The biotinylated and complementary rRNA probes were each precipitated using 0.1 volume 3 M Na-acetate (pH 5.2) and 2 vol. of 100% ethanol. The pellet was dissolved in RNase-free water. For a final clean up, an agarose gel-electrophoresis was performed with prior denaturation of the rRNA-probes using formamide^45^.

### rRNA depletion using biotinylated complementary rRNA probes

For capture of probes with bound rRNAs, streptavidin-coated magnetic beads (Dynabeads MyOne C1, Thermo Scientific) were used. The optimal amount of beads necessary for best removal of the biotinylated rRNA oligonucleotides was determined before (not shown). A volume of 225 µL magnetic beads (10 mg/mL) was found to give the best results when using 4 µg rRNA probes as follows. Beads were primed before use according to manufacturer and resuspended in 65 µL 2 × binding and wash buffer (10 mM Tris–HCl, 1 mM EDTA, 2 M NaCl).

All biotinylated complementary rRNA probes (5S, 16S, 5′ 23S, and 3′ 23S) were mixed in a total volume of 8 µL RNase-free water each with 2 µg absolute amount. In a volume of 40 µL, 2.5 µg total RNA isolated from the bacteria was mixed with 4 µL 10 × hybridization buffer (500 mM Tris–HCl, pH 7.5 and 1 M NaCl) and 8 µL mixture of the biotinylated rRNA-probes. Denaturation and hybridization were performed by incubation at 68 °C for 10 min, following 5 min at RT. For removal of the captured rRNAs, 65 µL primed magnetic beads were added and the mixture (105 µL) was vortexed for 10 s. Biotin-streptavidin binding was conducted for 5 min at RT and 5 min at 50 °C. Next, the magnetic beads were pulled by using rare-earth magnets in a magnetic rack for 1 min. The supernatant, with the remaining rRNA-depleted RNA, was transferred into a fresh tube and an ethanol precipitation was performed as before.

### rRNA depletion using commercially available kits

rRNA depletion was performed in triplicates of 2.5 µg total bacterial RNA for each kit tested. Depletion with the RiboZero Magnetic Kit for Bacteria (Illumina, discontinued November 2018), the RiboMinus Transcriptome Isolation Kit 1.0 (Yeast and Bacteria; Invitrogen), and MICROBExpress Kit (ambion, life technologies) were performed according to each manual. rRNA depletion with the riboPOOLs kit was performed according to the protocol v1.3 in the approaches for qPCR, quantity measurement by NanoDrop and Bioanalyzer and protocol v1.5 (for RNAs sequencing). As suggested by the manufacturer, Dynabeads MyOne Strep C1 (Thermo Scientific) were used. For all kits, the depleted RNA was precipitated with ethanol and resolved in 20 µL RNase-free water as above.

## Assessment of depletion efficiency

### Electropherogram

Depletion of rRNA was checked by the Agilent 2100 Bioanalyzer (Agilent Technologies) using the Agilent RNA 6000 Pico Kit according to the manual.

### Quantitative reverse-transcription PCR (qPCR)

Depletion of 5S and 16S rRNA was analysed by RT-qPCR (Supplementary table S6). Reverse transcription was performed with Superscript III (Thermo Scientific) using random nonamer primer (50 µM, Sigma Aldrich). Actual qPCR was conducted in triplicates using the SYBR Select Master Mix (Thermo Scientific) and 16 ng cDNA per reaction in a CFX96 Real Time System C100 Touch Thermal Cycler (BioRad). Primer efficiencies for 5S rRNA, 16S rRNA, and *cysG* (used as normalizer gene^46^), were determined using dilutions of genomic DNA (100, 50, 10, 5, 1, 0.5, and 0.05 ng of *E. coli* TOP10). The amount of remaining rRNA was determined using the ΔΔCt method^47^.

### RNA sequencing

The RNA was fragmented, dephosphorylated and re-phosphorylated to obtain all fragments irrespective of their initial phosphorylation status (e.g., native RNAs have a different phosphorylation status ^48^), which follows the procedure used by Hücker et al.^49^. Thus, 1 µg RNA is fragmented using a Covaris E220 (intensity, 175 W; time, 180 s; duty cycle, 10%; 200 cycles) in a Covaris-Tube (MicroTube AFA Fiber Pre-Slit –Snap-Cap 6 × 16 mm). After an ethanol precipitation, the RNA samples were resolved in 25 µL RNase-free water. Dephosphorylation was performed in 60 µl total volume by addition of 2.0 µL Antarctic Phosphatase (NEB), 2.7 µL 10 × Antarctic Phosphatase Buffer, 0.5 µL Superase·In (Invitrogen), and RNase-free water. Samples were incubated for 30 min at 37 °C and subsequently cleaned with the miRNeasy Mini Kit (Qiagen). The dephosphorylated RNA samples were dissolved in 30 µL RNase-free water. Phosphorylation was conducted by adding 3.6 µL T4-DNA-Ligase-Buffer (ThermoFisher), 2 µL T4 PNK (NEB), and 0.5 µL Superase·In and subsequent incubation for 1 h at 37 °C. The RNA was cleaned as before. Library preparation was performed according to the TrueSeq small RNA Kit (Illumina) manual. For size selection, polyacrylamide gels were prepared as follows. Per each gel, 4.2 mL MilliQ-water treated with DEPC, 2.475 mL Rothiphorese 30NF (29:1, Carl-Roth), 0.75 mL 10 × TBE, 75 µL 10% APS, and 4.5 µL TEMED were mixed and poured. Bands were cut after staining with SYBR Gold (Invitrogen) and comparison to a standard DNA ladder (1 kb-Plus, NEB) at a size range of about 27 to 40 bp. Final libraries were checked using an Agilent Bioanalyzer High Sensitivity DNA Analysis. Sequencing was performed on a MiSeq (Illumina) using a v3 cartridge for 2 × 75 cycles paired-end.

### Bioinformatics

The genomes files used for mapping were downloaded from NCBI (*E. coli* DSM 30,083, GCA_003697165.2*; E. coli* K-12 DH10B, GCA_000019425.1). Bioinformatic analysis of the raw sequencing reads was performed with a pipeline seamlessly connecting FastQC, Fastp, Bowtie2, and FastQ-Screen^50–53^.

## Results

### Self-made biotinylated probes (BP) for rRNA depletion

**Figure 1:**
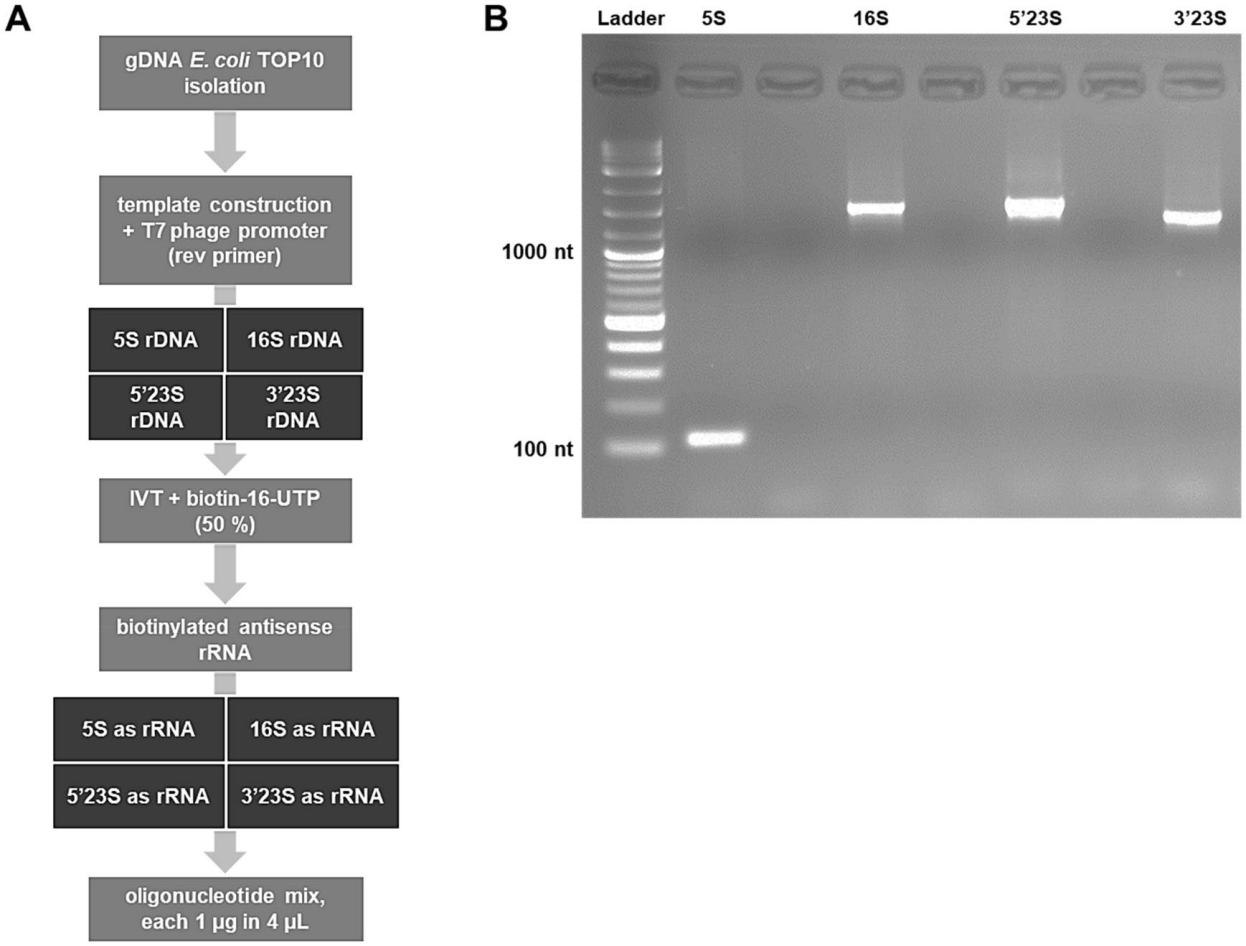
Construction of biotinylated antisense rRNA oligonucleotides. (**A**) Workflow for the construction of biotinylated antisense rRNA oligonucleotides. (**B**) Agarose-gel electrophoresis of biotinylated, full-length antisense rRNA oligonucleotides. Lanes have been labelled accordingly; ladder, 1 kb-Plus (NEB).

In order to accommodate the former RiboZero rRNA depletion kit, we produced biotinylated antisense rRNA probes for rRNA depletion. After rRNA and reverse probe hybridisation, the formed complexes are removed by using streptavidin-coated magnetic beads. In order to ease production of the full-length antisense rRNA probes, the probe for 23S rRNA was split, i.e., preparing the probes 5′ 23S and 3′ 23S. The other rRNAs, namely 5S and 16S rRNA, were covered in their entirety in a single product (Fig. 1A). Since RNA:RNA hybrids are more stable ^37^, it is advisable to indeed use complementary rRNA probes and not cDNA for depletion. The addition of a T7-promoter regions in reverse to the original rRNA genes by PCR and using an *in-vitro* transcription kit with a 1:1 mixture of UTPs and biotin-16-UTP makes the production of probes easy and straight forward (Fig. 1B). For the self-made version of the rRNA depletion protocol, we decided to use Dynabeads MyOne C1, which are streptavidin coated magnetic beads. Their specifications seem to fit best for our purposes, however, other such beads might also work. An advantage of the self-made version is the ability to customize the rRNA genes used. While the rRNA molecules in principle are similar in function and partly in sequence, differences exist (e.g., for bacteria with different GC values and / or strains far apart in taxonomy). Best binding efficiencies and, thus, rRNA removal, are assumedly achieved when using probes matching the target strain(s) well. Here, *E. coli* was used as test strain since this is the most common bacterium in the lab. In addition, *E. coli* had been used to develop depletions kits originally. Consequently, depletion kits should work best for this bacterium.

### Comparison between the different rRNA depletion kits, including the self-made version

**Figure 2:**
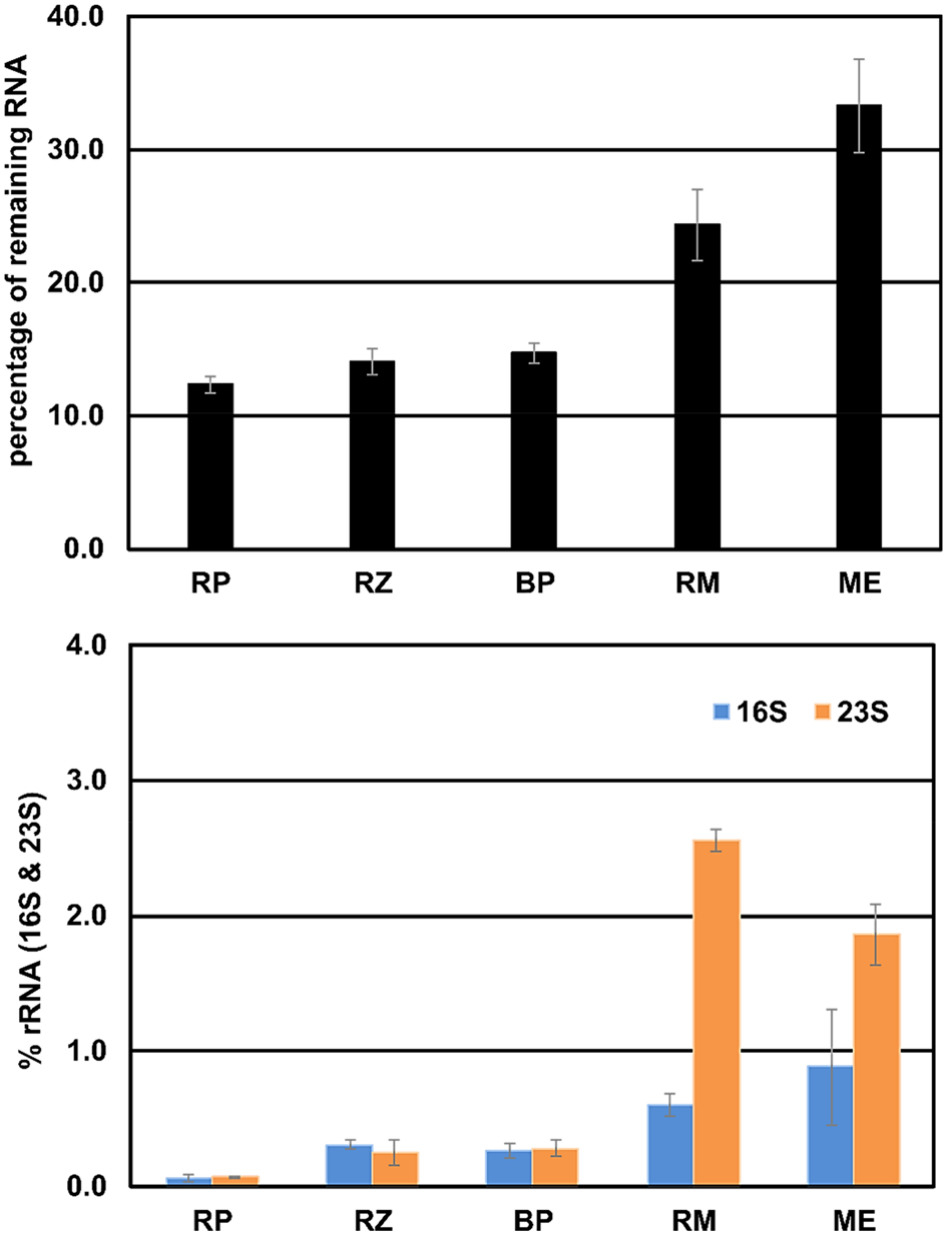
(**A**) Percentage of remaining RNA after depletion using the different depletion kits compared. The input of 2.5 µg total RNA was set to 100%. (**B**) Remaining 16S and 23S rRNA after depletion with the different kits compared to the untreated RNA determined by the Bioanalyzer electropherograms. RP, riboPOOLs; RZ, RiboZero; BP, biotinylated probes; RM, RiboMinus; ME, MICROBExpress.

The three commercially available rRNA depletion kits, e.g., RiboMinus (RM), MICROBExpress (ME), and riboPOOLs (RP) were compared to the discontinued old-version RiboZero (RZ) and the self-made depletion using biotinylated antisense rRNA probes (BP, see above). Depletions were performed in triplicates using an aliquot of the same total RNA isolated from *E. coli* in all cases*.* Depleted RNA was analysed quantitatively and qualitatively concerning the remaining RNA and efficacy of rRNA depletion. First, the remaining amount of RNA was measured using a Qubit (Fig. 2A). After depletion, the input amount of RNA was reduced to an output amount of below 40% for all approaches. ME showed an output RNA of about 33%, RM about 25%, while RP had an output of only 12% regarding to the input RNA, quite similar to RZ and the BP, both had about 14% RNA remaining. Since only about 10 to 20% of the total RNA is assumed to be non-rRNA, the depletion efficacy is already questionable concerning ME and RM since not as much material was removed. The latter two kits deplete only 16S and 23S rRNA, while RZ, RP and BP deplete all three rRNA species. However, simply comparing output versus input RNA after depletion does not specify which kind of RNA was removed or remained.

In a second step, specific depletion of 16S and 23S rRNAs was investigated using electrophoretic analysis (Fig. 2B). In the electropherograms, the relative amount of the rRNAs corresponds to the area under the curve for each rRNA peak. RP-treated samples have very little remaining 16S and 23S rRNA (below 0.1% each). In samples treated with RZ and BP, the remaining 16S and 23S rRNAs are below 0.5% each. For RM, small peaks for 16S and the 23S rRNA are visible correlating to 0.6% and 2.6% of 16S and 23S rRNA, respectively. In the ME depleted samples, the peaks of 16S and 23S rRNA correspond to 0.9% and 1.9%, respectively.

**Figure 3:**
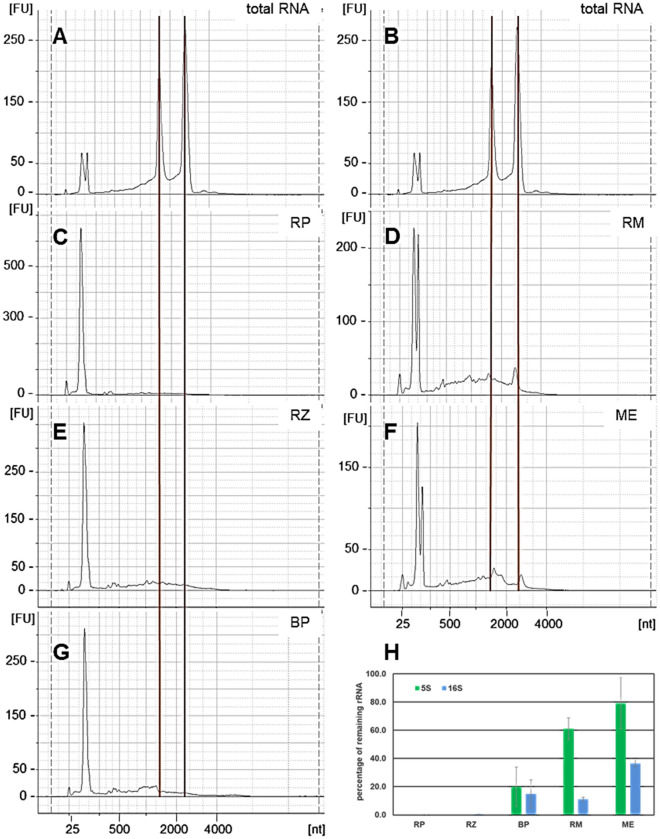
Electropherograms produced with the Bioanalyzer of RNA before and after depletion; the red lines indicate positions of 16S and 23S rRNA peaks, respectively. Total RNA untreated (**A**) RIN 8.9; (**B**) RIN 9.5. Both showing well-defined peaks of 16S and 23S rRNA signals. RNA depleted with (**C**) riboPOOL (RP); (**D**) RiboMinus (RM); (**E**) RiboZero (RZ); (**F**) MICROBExpress (ME); and (**G**) our biotinylated probes (BP). A strong decrease of the targeted rRNAs can be seen in the treated samples. Samples treated with RM and ME show some minor signals of remaining 16S and 23S rRNA. (**H**) Remaining amount of 5S and 16S rRNA after depletion, determined by qPCR. The amount of 100% was set for each quantified rRNA in the untreated total RNA.

Concerning 5S rRNA, the untreated total RNA shows a double peak in the beginning of each electropherograms, which might result from tRNA (about 80 nt) and 5S rRNA (about 120 nt). Two such samples of total RNA are shown in Fig. 3A,B. Similar to the untreated RNA, depletions using RM (Fig. 3D) and ME (Fig. 3F) still show a double peak since the 5S rRNA is not removed by these kits. In contrast, depletions using RZ, BP, and RP, which target the 5S rRNA, only one peak, e.g. the lower one, is found in the respective area indicating a reduction of 5S rRNA. The increase in height of the 5S rRNA/tRNA is due to the shift in composition in RNA-species after depletion. However, since 5S rRNA is close to tRNA in size, the remains of 5S was tested using qPCR (Fig. 3H). So far, we can summarize that RP, RZ and BP deplete 16S and 23S superior, while larger amounts especially of 23S rRNA remain when using ME or RM. In addition, the remaining larger peak at low molecular sizes in the electropherograms of RP, RZ and BP mainly consist of tRNAs, as shown when comparing to the qPCR results. Almost no 5S rRNA could be detected after RP and RZ depletion and only a low amount remained when depletion was performed using BP (Fig. 3H).

### Determination of remaining 5S and 16S rRNAs using qPCR

Since not all kits target and deplete 5S rRNA, the remaining amount of 5S and 16S present in each sample was measured using qPCR. The result was calculated for each method used based on the 5S and 16S rRNA amount in the untreated sample (Fig. 3H). The remaining amount of both rRNA species differed vastly. After using RZ and RP, negligible amounts of remaining 5S and 16S rRNA were found. BP removed both investigated rRNAs below 20%. Using RM, 10% of 16S rRNA remained and about 60% of the 5S rRNA compared to total RNA. Depletion with ME shows very high amounts of remaining 5S rRNA (80%) and 35% remaining 16S rRNA. While RM and ME do not target 5S rRNA at all, the observed reduction in 5S rRNA amount might be caused by unspecific removal of smaller RNAs.

### Quantitative analysis of remaining rRNAs using RNA-seq

**Figure 4:**
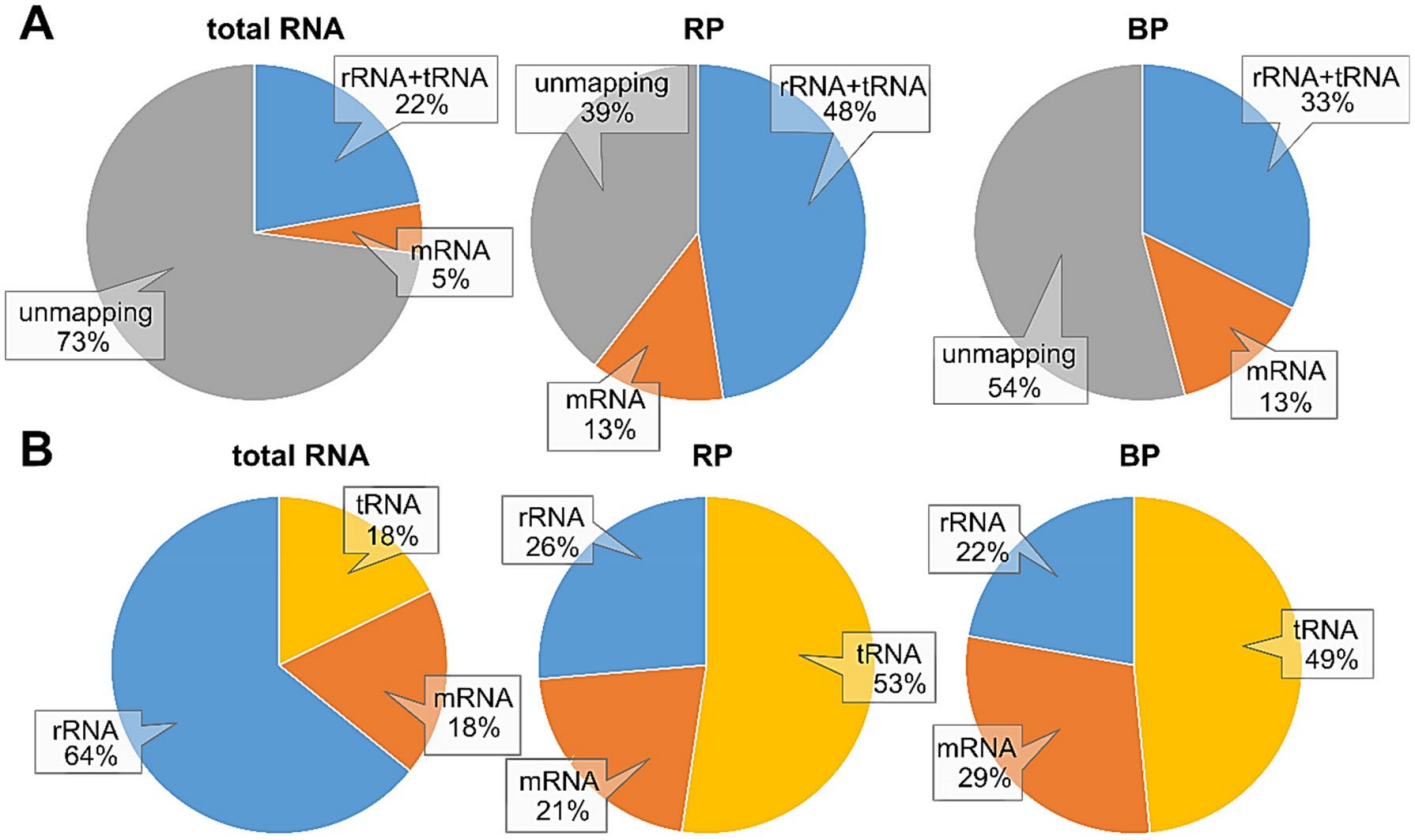
Distribution of reads mapping to the different RNA species or mapping not at all to the genome after RNA-seq. (**A**) Distribution of all raw reads, including those not mapping. (**B**) Distribution of the fractions of reads that map to the reference genome. Total RNA, untreated control sample; RP, sample treated with riboPOOLs; BP, sample treated with the biotinylated probes.

Sequencing data and, thus, final efficiencies in rRNA removal are available for RZ, RM and ME ^23,25–27^. However, RP and BP have not been tested with RNA-seq. Surprisingly, the number of reads that generally mapped to the genome increased after depletion. For instance, 27% of the reads from the untreated total RNA mapped to the genome, while 46% and 60% mapped when treated with BP and RP, respectively (Fig. 4A). Assumedly, the depletion reduces the overall amount of short rRNA fragments from degraded ribosomes. Such short reads only map with low confidence and are mostly excluded during bioinformatic analyses^54^. Nevertheless, after rRNA depletion, a 2.6-fold increase in reads mapping to protein-coding genes (i.e., derived from mRNA) was found. For the untreated total RNA about 5% mapped to genes, while about 13% of the reads mapped to genes for either treatment.

Comparing the distribution of the different RNA reads mapping to the genome, we found an increase for reads mapping to genes (i.e., originating from mRNA) and an even higher increase in reads mapping to tRNA. Nevertheless, whilst about 64% of the reads are mapping to rRNA genes in the untreated sample, a massive reduction of those reads is seen after treatment, e.g., about 26% for RP and about 22% for BP map to rRNA genes. This reduction in rRNA-originating reads leads to a shift towards mRNA- and tRNA-originating reads, which are therefore increased in proportion (Fig. 4B).

### Comparing relative mRNA reads

**Figure 5:**
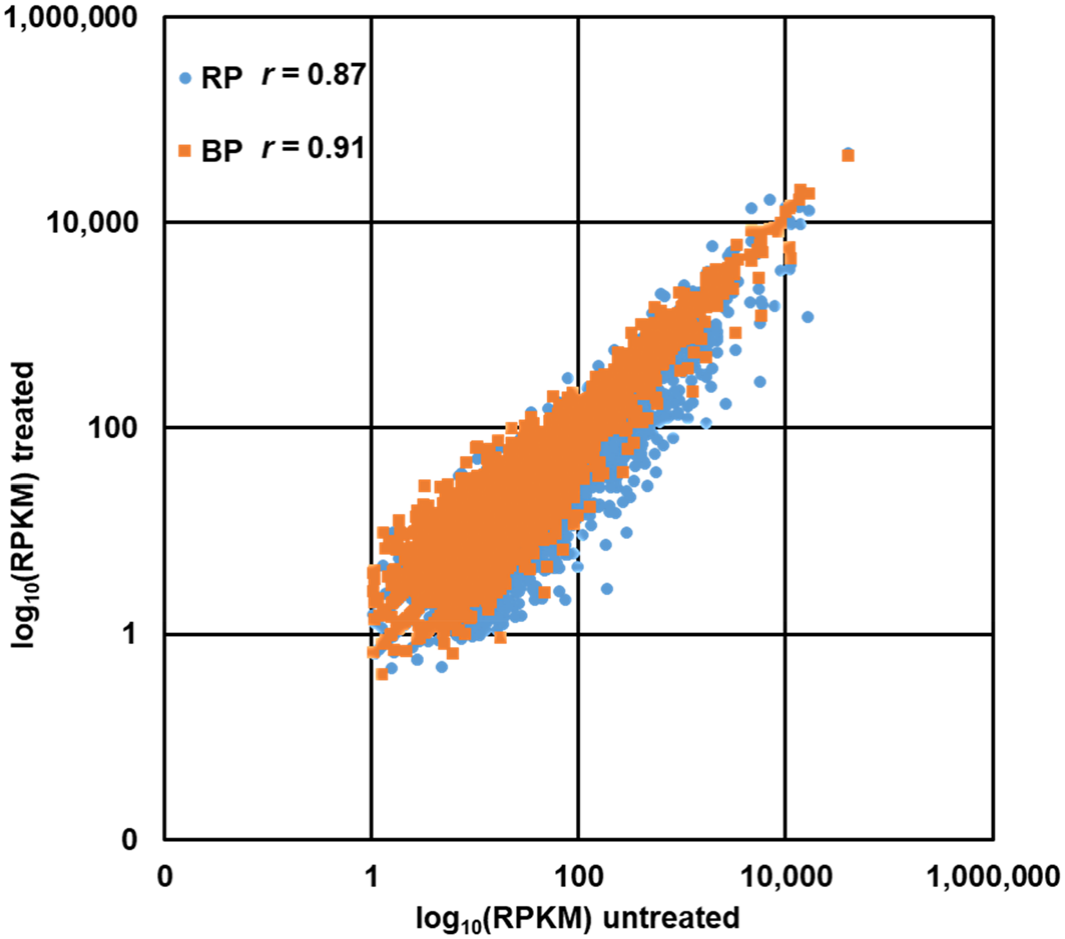
Correlation between log_10_ CDS (coding sequence)-mapping RPKM for libraries depleted with RP (blue) and BP (orange), compared to untreated RNA. Fragments of all lengths were included for computing RPKMs. Pearson correlations are indicated.

For all three RNA-seq experiments conducted here, the very same total RNA was used as raw material. Therefore, we expected the relative mRNA abundances should be the same for untreated and for BP- or RP-treated samples. The Pearson correlation of RPKM (reads per kilo base per million mapped reads) values for gene-coding regions (CDS) were 0.91 and 0.87 when comparing untreated RNA with the sample depleted with BP and RP, respectively (Fig. 5, Supplementary Fig. S1–S2). The Pearson correlation between BP and RP is 0.89 (Fig. S3), indicating a strong positive association. Thus, we found no substantial difference in expression levels for CDS between the two depletion approaches. Further, read counts mapping to gene-coding regions were normalized as RPM (thus, not normalized to the gene length) and compared for untreated RNA with depleted RNA (Fig. S4). The Pearson correlation of the RPM data of treated and untreated RNA shows similar values as when comparing RPKM data, untreated vs. RP has a correlation of 0.89 and untreated vs. BP a correlation of 0.97. The greater variance observed between RP and the untreated sample compared to a lesser variance between BP and the untreated sample, indicating a larger bias in the former.

**Figure 6:**
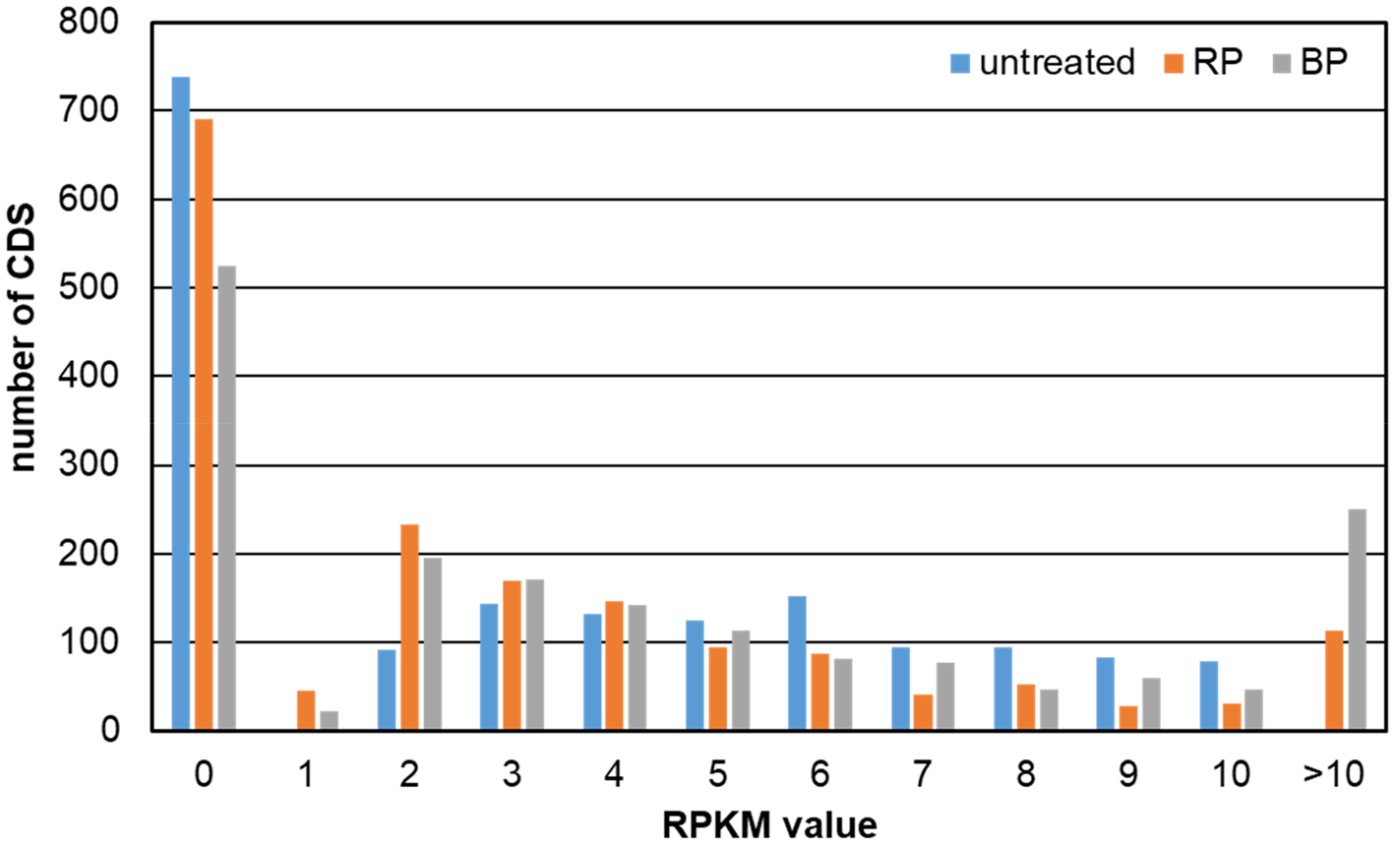
Distribution of 1731 CDS below RPKM 10 in the untreated sample compared to their RPKM distribution in the rRNA-depleted samples, either using RP or BP.

In contrast to the bulk of genes, comparing weakly expressed genes (i.e., RPKM ≤ 10) in the untreated and treated sample shows a much lower correlation. For instance, the Pearson correlation of such weakly expressed genes comparing the untreated sample with RP and BP is 0.28 and 0.33, respectively. However, this is not unexpected since the detection of a single read more or less reflects a substantial percent change for genes with such low expression values. In any case, in the untreated sample, 1731 protein-coding genes with an RPKM at or below 10 were found. Interestingly, the fraction of genes with no observable expression (i.e., RPKM = 0) is higher in the untreated sample (e.g., 738), but lower in the treated samples, e.g., 690 and 524 for RP and BP, respectively. Thus, depletion increases the number of genes displaying detectable expression at all. Furthermore, 113 and 251 genes formerly having an RPKM ≤ 10, are now above 10 RPKM when the total RNA was depleted with RP with BP. Taken together, a general shift of more observable reads per gene for the weakly expressed genes is visible after depletion (Fig. 6). Of note, the observed lesser number of genes between 5 and 10 RPKM in Fig. 6 after depletion is virtual, since most of those “missing” genes are moved to the fraction of genes with RPKM above 10. Similarly, the increased abundance of genes with RPKM values between 1 and 4 after depletion is due to a shift of genes from those with 0 reads before.

## Discussion

The differences between the efficiency of rRNA depletion of different methods tested here seem to be caused by the characteristics of their specifications. Comparing results received by RT-qPCR and Bioanalyzer data, a clear ranking of depletion efficiency can be made with RP reaching comparable results as the former RZ. Our own biotinylated probes seem to be a little less efficient compared to RP or RZ, but all of the former are more efficient than RM or ME. One main reason for the differences observed might be that RP, RZ and BP target 5S, 16S and 23S rRNAs, whereas RM and ME targeted 16S and 23S rRNA only. Despite, RM and ME are also inferior in depleting the targeted 16S and 23S rRNAs. Perhaps, this is due to the short oligonucleotides used in both kits of just around 20 nt. Additionally, it is unclear if the complete sequence of the rRNAs is covered by those probes. Hence, small fragments of rRNA, which may appear due to degradation, might not be captured efficiently and remain in the samples. Further, the hybridisation efficiency of oligonucleotides is inferior compared to longer probes. For instance, the antisense oligonucleotides used in RP are 40 nt and presumably cover the full length of each rRNA species. This increased length in the probes results in better depletion, even if degradation of rRNAs occurs. The nucleotides used in RZ and in the BP method are full-length antisense molecules of the rRNAs and are RNA-based in contrast to the (c)DNA-based probes used in RM, RP, and ME. Additionally, RNA:RNA hybrids are more stable than RNA:DNA hybrids ^37,38,55^. Therefore, we can assume that the hybridisation is faster, the hybridisation complex is more stable, and depletion is more efficient for RZ and BP. Depletion efficacies of RZ, RM and ME were already investigated before. Bhagwat et al. (2014a, b) describes similar efficiencies for the depletion of rRNAs from *Salmonella* species as we found here for *E. coli* ^56^. Also, Ciulla et al.^23^, comparing RZ, ME and three further depletion setups with other strategies, showed that RZ was superior in depletion of all rRNAs at that time. Petrova et al.^26^ compared RZ, ME and RM using total RNA obtained from a co-culture of *Pseudomonas aeruginosa* and *Staphylococcus aureus*^7,23,26^*.* Their results regarding depletion efficiency of rRNAs with RZ, RM and ME were confirmed by our data.

Our further investigations focused in BP and RP compared to untreated RNA. After comparing the relative mRNA reads from RNA-seq, differences between the untreated and the treated samples for both methods are observed. The varying numbers of gene-coding reads can be traced back either to varying degradation of some mRNAs during preparation or, more likely, to varying unspecific depletion of certain mRNA during the rRNA depletion. Nevertheless, our data shows a good correlation between treated and untreated samples and also an increasing amount of effective reads deriving from mRNA, especially from low expressed genes. For those genes, a shift to higher RPKM values is observed. Thus, the reduction of rRNA molecules increases their detection potential.

Taken together, the self-made BP has a similar depletion efficiency compared to the former RZ (Figs. 2, 3). In addition, we assume a similar performance since the basic principles between BP and RZ are the same. The probes used for BP cover the complete rRNA sequences, which was also the case for RZ. Thus, even fragments of degraded rRNA molecules can be depleted. As said, using antisense RNA allows a more efficient rRNA removal. Further, the probes contain biotin at several positions in their sequence. This increases the probability of binding to the magnetic beads also in cases of degraded probes. In other experiments, when not using full-length probes, we found higher amounts of probe sequences appearing antisense to the rRNA genes in the final data (data not shown).

Finally, the BP-method can be customized to the target organism or target RNA. It might even be possible to derive antisense tRNA probes, which allow tRNA removal, as mentioned by Liu et al.^57^, but this is not commercialized yet. tRNA depletion would be a valuable target for depletion, since we found a high proportion of tRNAs present in the samples after depletion. Nevertheless, a possible disadvantage of the BP method is the somewhat lower stability of the RNA probes. In any case, also the RNA samples have to be treated with the same care in order not to degrade precious samples. Unfortunately, it was not possible to compare RNA-sequencing results of a RZ depleted probe concerning expression levels, since the former RZ kit is not available anymore. However, from our previous data (not shown) and experiments conducted here, we conclude a very similar overall performance.

If the user prefers a ready-to-use product, RP is a good alternative to the former RZ, with comparable efficiencies and modest bias in changing gene expression values due to the depletion procedure. The new version of RZ is enzyme based. It should be treated with some care, especially when comparing datasets derived from using the previous version with data from using the new version. It has been shown that enzyme-based kits lead to a larger bias, especially in Ribo-seq experiments ^28,54^.

All tested kits/methods are similar in price per reaction (~ 60 €/ reaction, Supplementary table S1). While some researchers recommend performing ribosomal profiling without any depletion of rRNA, we found that BP is a straightforward method for depletion increasing the amount of effective reads and cutting final costs.

## Conclusion

In our hands, RP turned out to be a good alternative concerning the old version of the RZ, showing comparable results. Further, specific depletions can be conducted. Our method, BP, which basically is a “bootlegged” version of the old RZ also showed sufficient depletion. Creating biotinylated probes is straightforward when using gDNA of the organisms of interest. Even further, any sequence of interest and not only rRNA could be used for capturing and removal. The new version of RZ, using RNase H, must be evaluated in the future. Data for the comparison between hybridisation removal and magnetic-bead capture or RNase H digestion are still yet missing. Anyway, we (highly) recommend using hybridization and capture methods for rRNA depletion in RNA-seq or Ribo-seq experiments. The shift in RNA species composition increases clearly the amount of mRNA reads mapping and additionally, improves detectable expression for weakly expressed genes.

## Supplementary Information

Supplementary Information.
