# Supplementary material for: Comparison of rRNA depletion methods for efficient bacterial mRNA sequencing
Source: Sci Rep. 2022 Apr 6;12:5765. doi: 10.1038/s41598-022-09710-y (PMC8986838; doi:10.1038/s41598-022-09710-y)

**Comparison of rRNA depletion methods for efficient bacterial mRNA sequencing**

Anika Wahl ^1,2^, Christopher Huptas ^1^, Klaus Neuhaus ^2, *^

^1^ Chair for Microbial Ecology, Technische Universität München, Weihenstephaner Berg 3, 85354 Freising

^2^ ZIEL Core Facility Microbiome, Technische Universität München, Weihenstephaner Berg 3, 85354 Freising

^*^ corresponding: neuhaus@tum.de

**List of abbreviations**

**Abbreviation Full descriptions**

as antisense

BP biotinylated probes

DNA deoxyribonucleic acid

cDNA copy DNA

gDNA genomic DNA

CDS coding sequence

CTAB cetyltrimethylammonium bromide

IVT in vitro transcription

ME MICROBExpress

PCR polymerase chain reaction

Ribo-seq ribosomal profiling

RM RiboMinus

RNA ribonucleic acid

RNA-seq RNA sequencing

RPKM reads per kilo base per million mapped reads

RPM reads per million

RT-qPCR reverse transcriptase quantitative PCR

RZ RiboZero

UTP Uridine triphosphate

**Supplementary data**

**Supplementary tables S1 – S6**


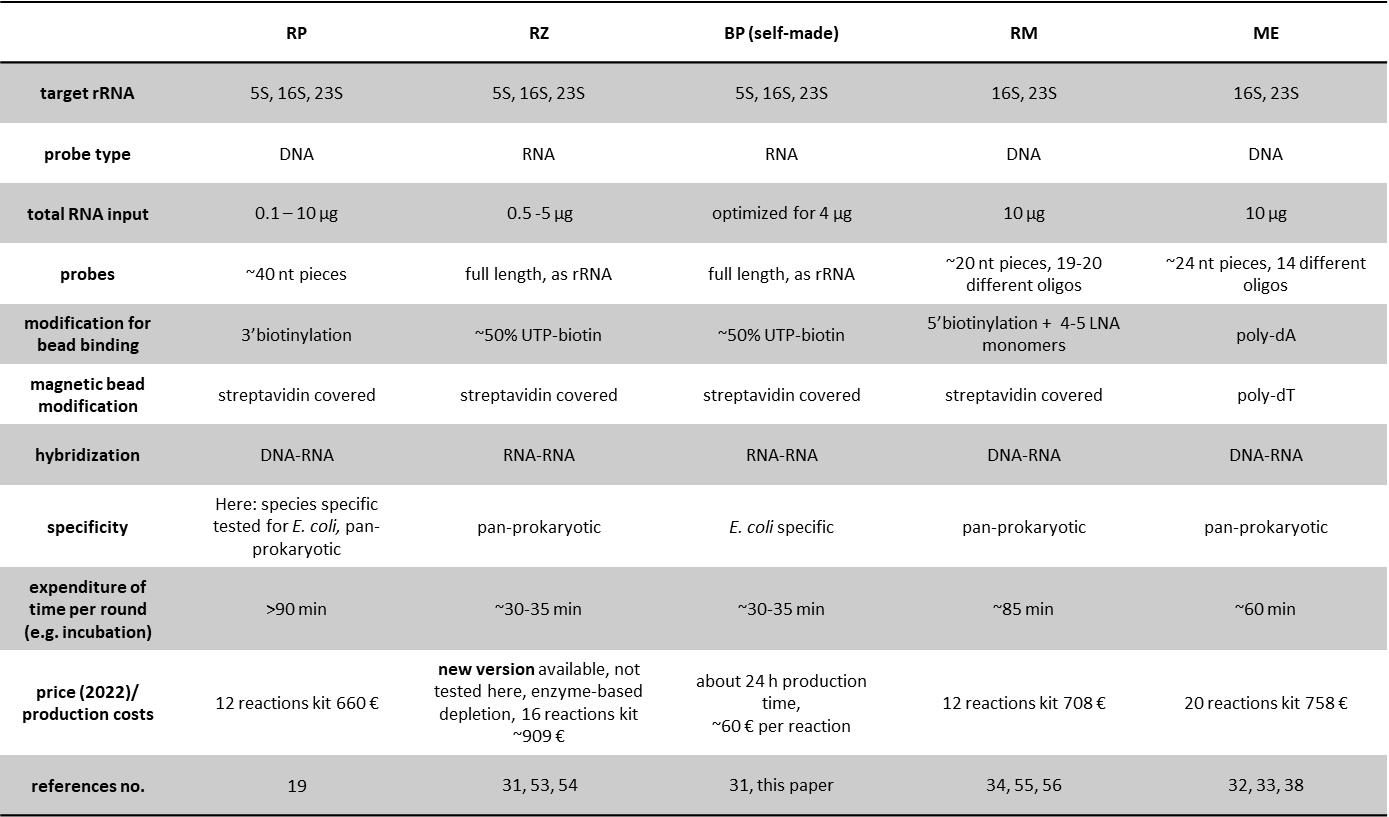
**Table S1.** Comparison of the different commercially available depletion kits


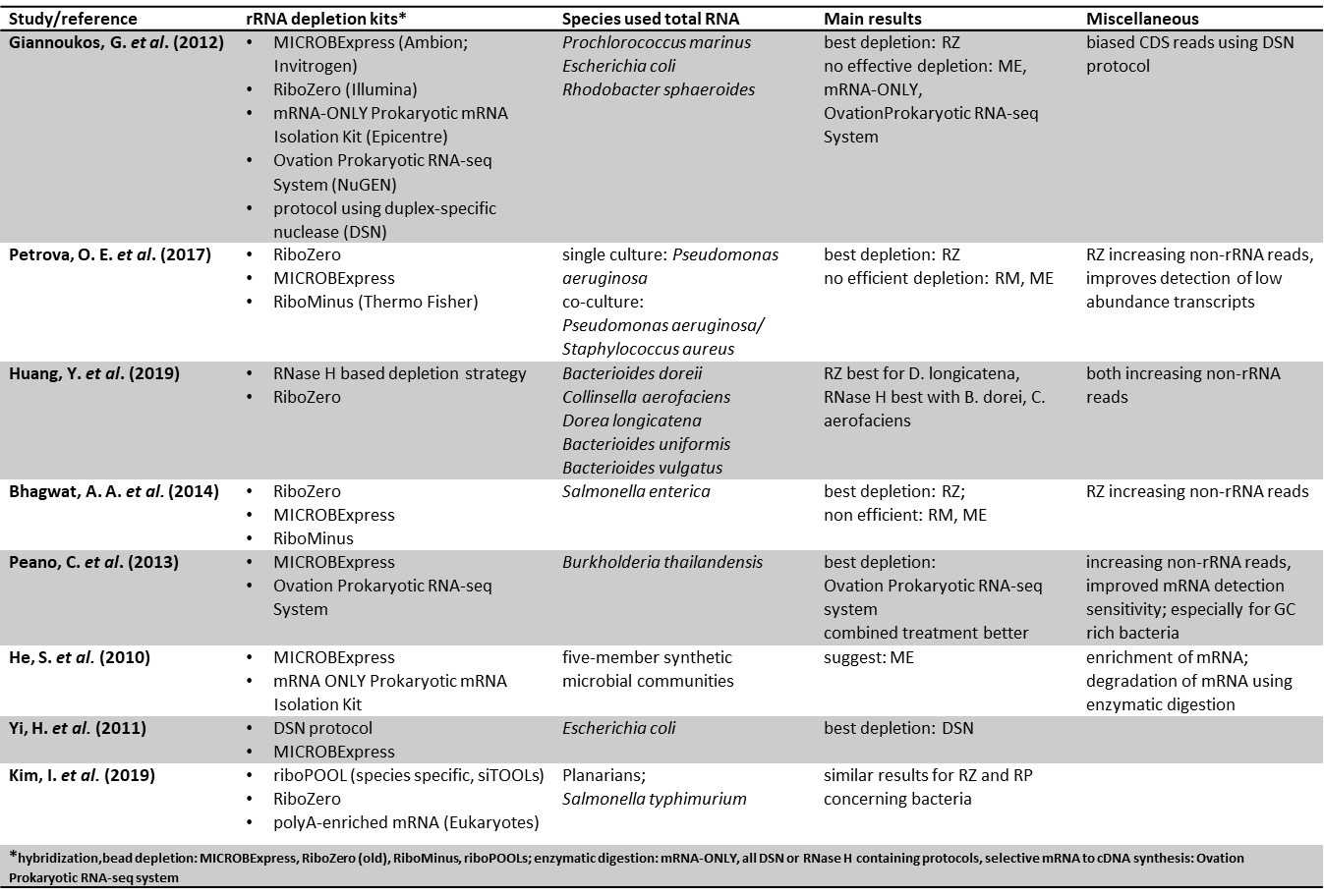
Table S2. Summarized literature review of different depletion kits

Table S3. List of primer

| **Primer** | **Sequence 5‘🡪3‘** |
| --- | --- |
| 5SrDNAfw | TGCCTGGCGGCAGTAGCGCGGT |
| 5SrDNArevT7P | AATTCTAATACGACTCACTATAGGGAGATGCCTGGCAGTTCCCTACTCTC |
| 16SrDNAfw | AGAGTTTGATCCTGGCTCAG |
| 16SrDNArevT7P | AATTCTAATACGACTCACTATAGGGAGAGGAGGTGATCCAACCGCAGGTT |
| 5'23SrDNAfw | AAGCGACTAAGCGTACACGGTGGA |
| 5'23SrDNArevT7P | AATTCTAATACGACTCACTATAGGGAGATTCCTGGAAGCAGGGCATTTGTTG |
| 3'23SrDNAfw | CAACAAATGCCCTGCTTCCAGGAA |
| 3'23SrDNArevT7P | AATTCTAATACGACTCACTATAGGGAGACACGGTTCATTAGTACCGGTTAGCT |
| q-cysGfw | TTGTCGGCGGTGGTGATGTC |
| q-cysGrev | ATGCGGTGAACTGTGGAATAAACG |
| q-5SrDNAfw | TGCCTGGCGGCAGTAGCGCGGT |
| q-5SrDNArev | TGCCTGGCAGTTCCCTACTCTC |
| q-16SrDNAfw | GAGGTGATCCAACCGCAGGTT |
| q-16SrDNArev | AGAGTTTGATCCTGGCTCAG |
| rrsHR | GGAGGTGATCCAACCGCAGG |
| rrsHF | AATGTTGGGTTAAGTCCCGC |

**Table S4.** PCR with *Taq* DNA polymerase

| **Reaction mixture (25 µl)** | | **Reaction conditions (30 cycles):** | |
| --- | --- | --- | --- |
| 10x PCR buffer | 2.5 μL | Initial denaturation | 95 °C 5 min |
| 10 mM dNTP | 1 μL | Denaturation | 95 °C 30 s |
| 10 μM forward primer | 1.25 μL | Annealing | 55 °C 30 s |
| 10 μM reverse primer | 1.25 μL | Elongation | 68 °C 30 s |
| *Taq* DNA polymerase | 0.125 μL | Final elongation | 68°C 5 min |
| template gDNA/RNA | 100 ng |  |  |
| water | add. 25 µL |  |  |

Table S5. PCR with Q5 high-fidelity polymerase

| **Reaction mixture (25 µl):** |  | **Reaction conditions (30 cycles):** | |
| --- | --- | --- | --- |
| 5x Q5 buffer | 5 µL | Initial denaturation | 98 °C 3 min |
| 5x GC buffer | 5 µL | Denaturation | 98 °C 10 s |
| 10 mM dNTPs | 0.5 µL | Annealing | 68 °C 30 s |
| 10 µM forward primer | 1.25 µL | Elongation | 72 °C 20 s |
| 10 µM reverse primer | 1.25 µL | Final elongation | 72 °C 2 min |
| Q5 high-fidelity polymerase | 0.25 µL |  |  |
| template | 1 µL |  |  |
| water | add. 25 µL |  |  |

Table S6. qPCR conditions

| **Reaction mixture (25 µl):** |  | **Reaction conditions (42 cycles):** | |
| --- | --- | --- | --- |
| SYBR select master mix | 12.5 µL | Initial denaturation | 95 °C 3 min |
| 50 µM forward primer | 0.5 µL | Denaturation | 95 °C 20 s |
| 50 µM reverse primer | 0.5 µL | Annealing | 60 °C 30 s |
| template cDNA/gDNA | 2 µL | Elongation | 72 °C 40 s |
| water | 9.5 µL | Final elongation | 72 °C 2 min |

**Supplementary Figures S1 – S4**

Figure S1. Correlation between CDS reads in un-treated RNA and BP-treated RNA. The correlation is represented with the log_10_ CDS-mapping RPKM for the libraries. The calculated Pearson correlation is 0.91.


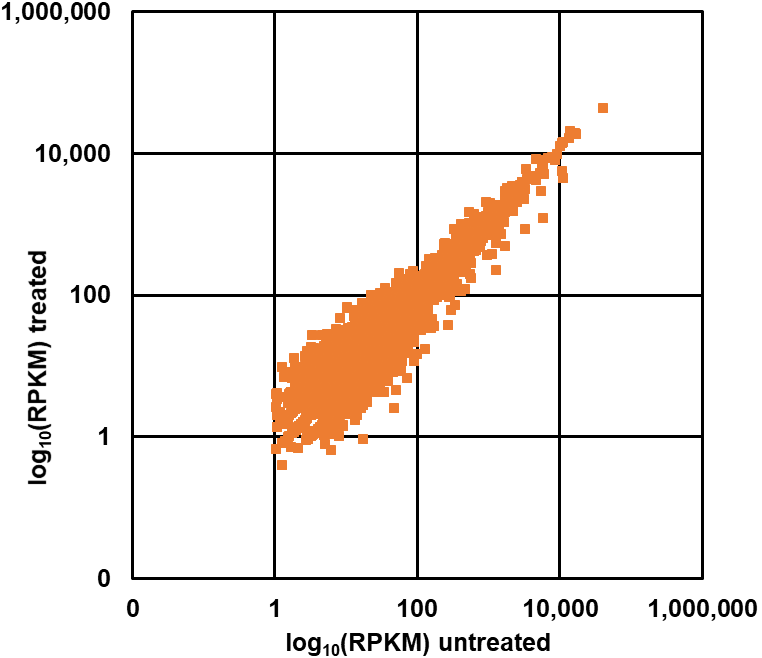


Figure S2. Correlation between CDS reads in un-treated RNA and RP-treated RNA. The correlation is represented with the log_10_ CDS-mapping RPKM for the libraries. The calculated Pearson correlation is 0.87.


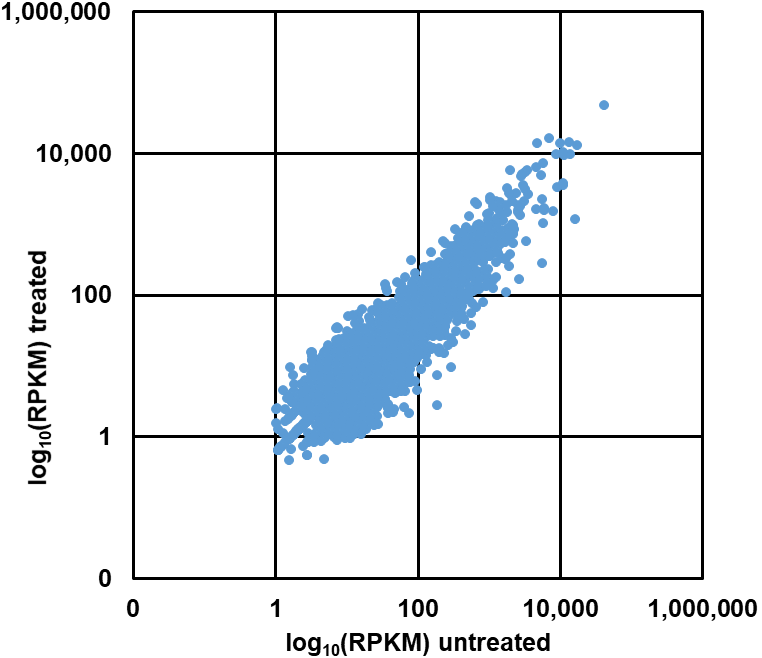


Figure S3. Correlation between CDS reads in BP-treated RNA and RP-treated RNA. The correlation is represented with the log_10_ CDS-mapping RPKM for the libraries. The calculated Pearson correlation is 0.89.

Figure S4. Correlation between CDS reads in un-treated RNA and RP/BP-treated RNA. The correlation is represented by the RPM. The comparison of BP-treated RNA with un-treated RNA is coloured in orange, for RP-treated RNA in blue. The Pearson correlation is indicated in the legend.


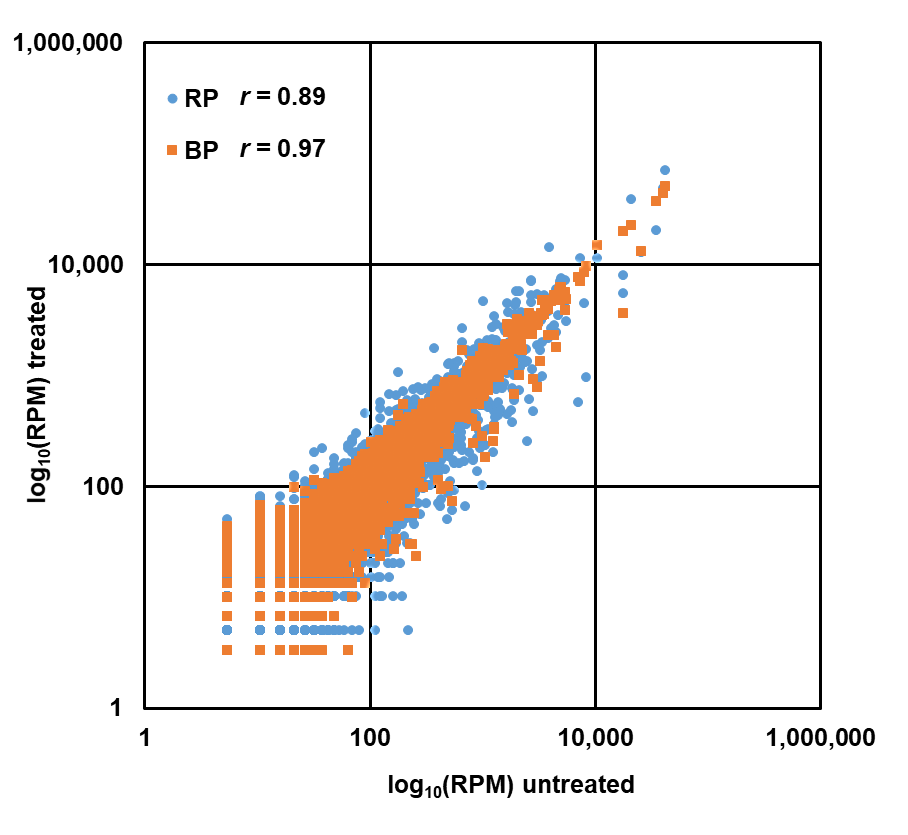

Supplement: Supplementary file 1 — Supplementary Information. [file 41598_2022_9710_MOESM1_ESM.docx]
